# World Heart Federation Briefing on Prevention: Coronavirus Disease 2019 (COVID-19) in Low-Income Countries

**DOI:** 10.5334/gh.778

**Published:** 2020-04-09

**Authors:** Friedrich Thienemann, Fausto Pinto, Diederick E. Grobbee, Michael Boehm, Nooshin Bazargani, Junbo Ge, Karen Sliwa

**Affiliations:** 1 Hatter Institute for Cardiovascular Research in Africa and Department of Medicine, Faculty of Health Sciences, University of Cape Town, ZA; 2 Department of Internal Medicine, University Hospital Zurich, University of Zurich, CH; 3 World Heart Federation, Rue de Malatrex 32, 1201 Geneva, CH; 4 Santa Maria University Hospital, CAML, CCUL, Faculdade de Medicina da Universidade de Lisboa, Lisbon, PT; 5 Julius Global Health, the Julius Center for Health Sciences and Primary Care, University Medical Center Utrecht, NL; 6 University of Saarland, Homburg, DE; 7 葛均波 ZS-hospital, Shanghai, CN

**Keywords:** coronavirus, COVID-19, Africa, low income countries, middle income countries, cardiovascular disease

## Abstract

In December 2019, the novel coronavirus Coronavirus Disease 2019 (COVID-19) outbreak started in Wuhan, the capital of Hubei province in China. Since then it has spread to many other regions, including low-income countries.

In December 2019, the novel coronavirus *Coronavirus Disease 2019 (COVID-19)* outbreak started in Wuhan, the capital of Hubei province in China. Since then it has spread to many other regions, including low-income countries [1,2]. The coronavirus was named SARS-CoV-2 (Severe Acute Respiratory Syndrome Coronavirus 2) and has spread to 78 countries (including many low-income countries), with a total of 92,818 confirmed cases globally as of 03 March 2020 (Figure 1). On 30 January 2020, the World Health Organization (WHO) declared the outbreak a ‘public health emergency of international concern’. The first ten cases have now been reported in Africa (Algeria, Egypt, Morocco, Nigeria and Senegal). The spread onto the African continent is of great concern for multiple reasons [3]. Large and densely populated areas and townships with widespread poverty and high migration are the most vulnerable populations for airborne pandemics. Moreover, existing epidemics of human immunodeficiency virus (HIV), tuberculosis (TB) and malaria are likely to collide with COVID-19 and may lead to an increased morbidity and mortality – not reported yet from affected countries. In addition, the wide spread of non-communicable diseases in Africa, such as chronic obstructive pulmonary disease (COPD), heart disease, hypertension and diabetes are known risk factors for severe causes of COVID-19 [2].

**Figure 1:**
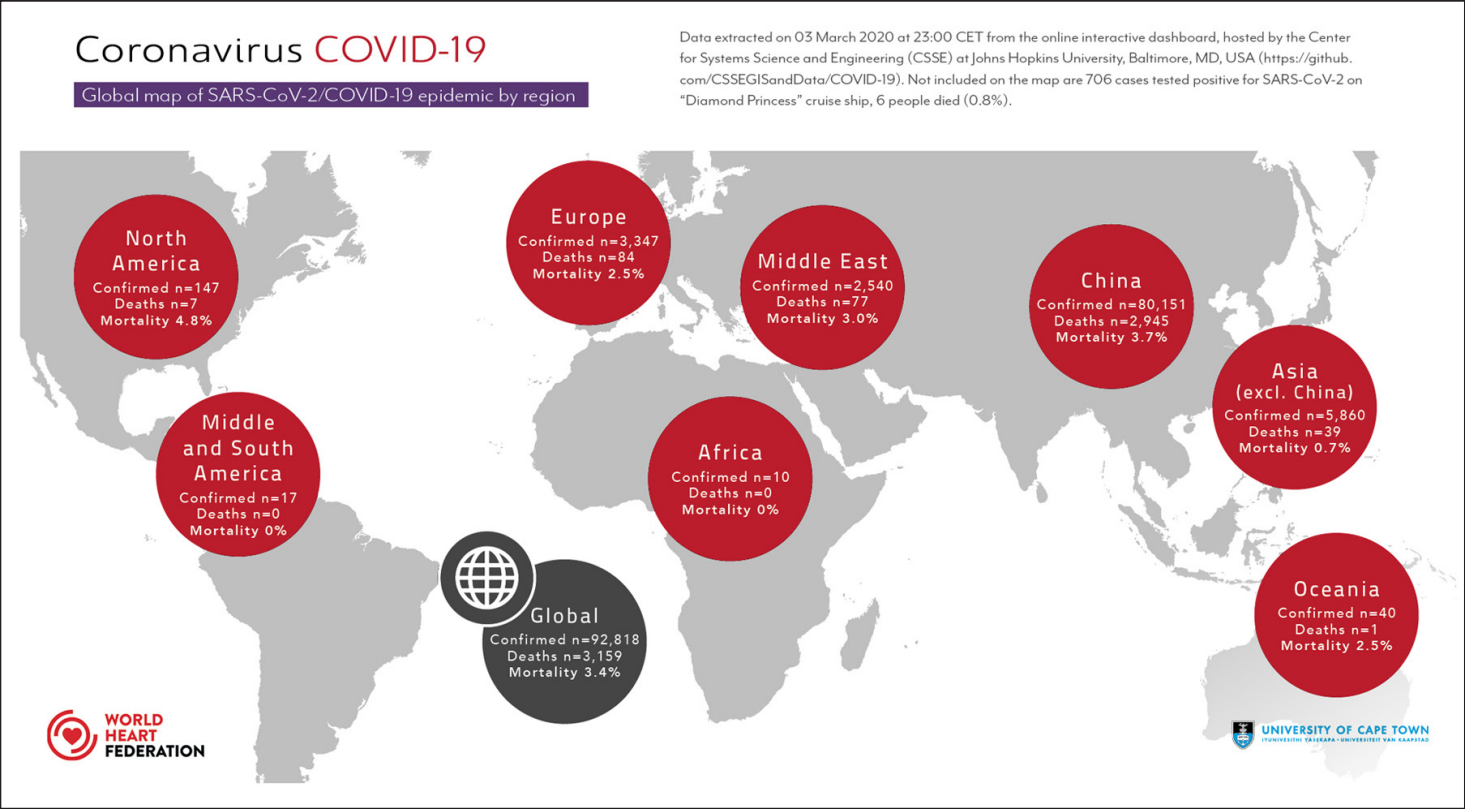
Global map of SARS-CoV-2/COVID-19 epidemic by region.

During the past 20 years, outbreak and prevalence of severe respiratory infections have been seen as a major hazard to global health. In December 2019, a series of pneumonia cases of unknown aetiology were documented in Wuhan. High-throughput sequencing from respiratory tract samples revealed this novel coronavirus strain, named SARS-CoV-2 [4]. In response to this outbreak, most areas in China have initiated policies to restrict access and traffic, as well as other measures according to the national prevention and control plan. China’s strong response to containing the COVID-19 epidemic was best practice and exemplary to the world. Back in 2003, there were 305 cases (including five deaths) caused by the outbreak of Severe Acute Respiratory Syndrome (SARS-CoV) before the Chinese government reported it to the WHO on 10 February 2003. However, there were only 27 cases (and no deaths) due to COVID-19 before it was reported to the WHO in January 2020. Since then, the global clinical and scientific community has established guidelines for prevention, diagnosis and management and is working continuously on therapeutic compounds and vaccines.

As a global organization representing the cardiovascular community, with more than 200 cardiovascular societies and foundations, the World Heart Federation is concerned that previous studies on other coronavirus diseases such as SARS-CoV and MERS-CoV have demonstrated a relationship between cardiovascular disease (cardiac disease and hypertension), diabetes and an increased morbidity and mortality due to coronavirus disease [5,6]. Guan et al. reported in the New England Journal of Medicine on a case series of 1,099 cases with COVID-19 admitted to hospital in China. Patients with co-morbidities such as COPD, coronary artery and cerebrovascular disease, hypertension and diabetes were more likely to develop severe COVID-19 disease compared to patients without co-morbidities [2]. Another unpublished case series reported that about 10% of COVID-19 with severe disease developed acute cardiac injury with raised troponin-I/T (Bo Li et al, Clinical Research Cardiology, 2020, in press).

The goal of this document is to update our members from low-income countries on important facts on COVID-19 prevention in low- and middle-income countries.

## Important facts

### 1. The novel coronavirus

Coronaviruses belong to a family of viruses that can cause mild disease such as a common cold, but also severe respiratory disease such as Middle East Respiratory Syndrome (MERS-CoV) or Severe Acute Respiratory Syndrome (SARS-CoV). The novel coronavirus epidemic Coronavirus Disease 2019 (COVID-19) is caused by a coronavirus named SARS-CoV-2 (Severe Acute Respiratory Syndrome Coronavirus 2).

### 2. Transmission and infection

**Figure 2:**
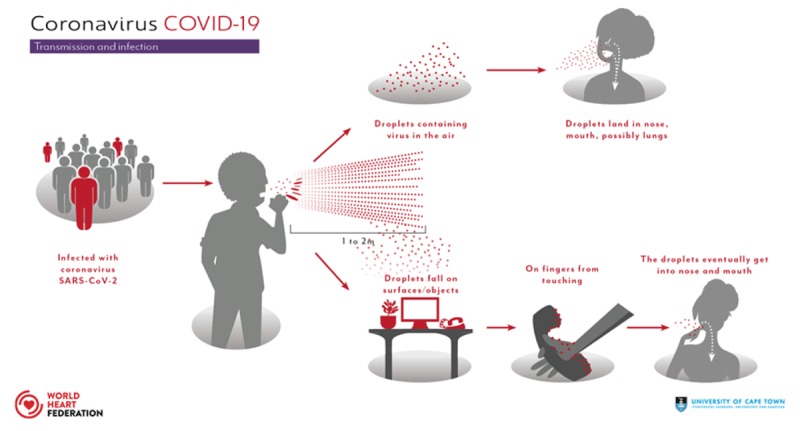
Transmission and infection.

SARS-CoV-2 is thought to have a zoonotic origin and was first isolated from patients with pneumonia in Wuhan, China. The spread from human-to-human is mainly an aerosol transmission through contaminated respiratory droplets (coughing and sneezing). In addition, respiratory droplets containing the virus may contaminate surfaces up to 96 hours, for example screens of smart phones (Figure 2).

### 3. Signs and symptoms

**Figure 3:**
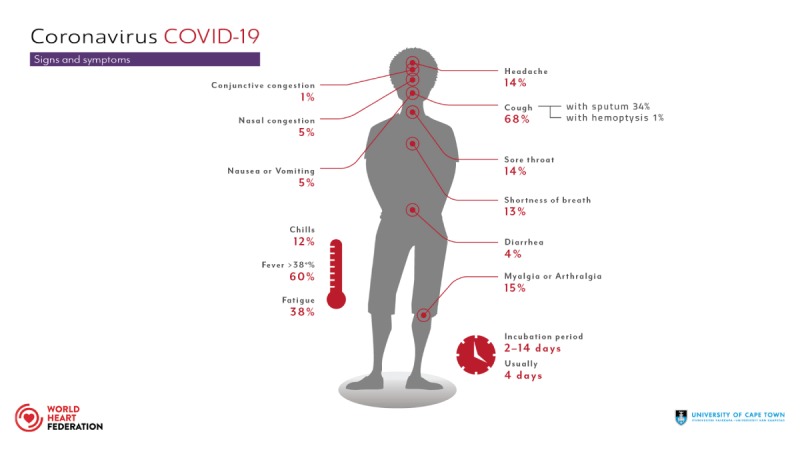
Signs and symptoms.

Signs and symptoms of COVID-19 include flu-like symptoms such as fever, cough, fatigue, headache, sore throat, shortness of breath and myalgia. In rare cases nausea, vomiting and diarrhoea have occurred. In severe cases pneumonia, acute respiratory distress syndrome (ARDS), multi-organ failure and death have been reported (Figure 3).

### 4. Prevention

**Figure 4:**
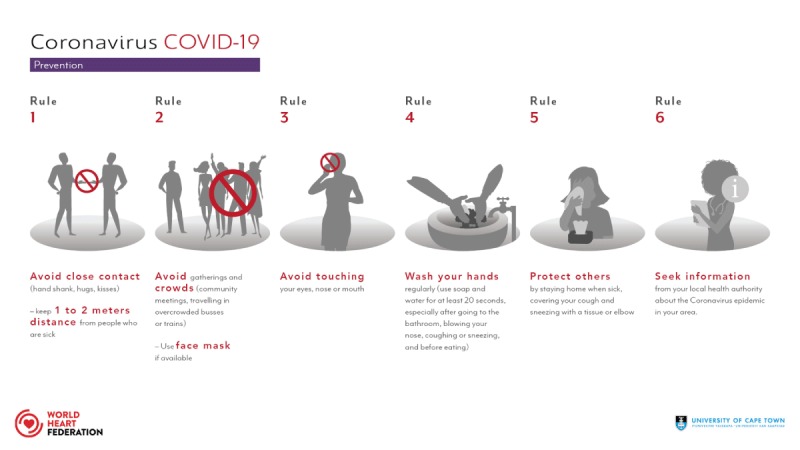
Prevention.

Basic recommendations to prevent COVID-19: Follow six easy rules to reduce the risk of coronavirus transmission. The rules are built on the principle of protecting yourself and protecting others (Figure 4).

## Recommendations on prevention for special populations

People with chronic underlying disease may be at increased risk of severe COVID-19 disease and death (Table 1). In the largest Chinese cohort, 16% of patients developed severe disease with a mortality rate of 8.1% [2]. Of those patients with severe disease, 38.7% had co-morbidities. Therefore, patients with co-morbidities require more rigorous prevention mechanisms. For people with chronic respiratory disease (e.g. chronic obstructive airways disease), infectious diseases (e.g. HIV and tuberculosis), chronic cardiovascular disease (e.g. cardiomyopathy, previous myocardial infarction, rheumatic heart disease), cancer or autoimmune diseases we recommend:

1. Avoid large gatherings – stay at home.
2. Keep at least 1–2 meters distance from a person with respiratory symptoms and do not stay in the same room with this person.
3. Vulnerable people should consider moving to relatives in rural areas and spend their time in voluntary isolation, such as a small hut, receiving food supplies via a neighbour or relative, but without direct contact.
4. Travel should be reduced to a minimum. Use a mask if travelling in a bus, train or plane. If masks are not available or affordable, cover your nose and mouth with a cloth or similar.

**Table 1:** Risk factors for severe disease.

Risk factors for severe disease	

1. Age	>52 years (interquartile range 40–65)
2. Co-morbidities present in 38% of patients with severe disease	Hypertension 24% Diabetes 16% Coronary heart or cerebrovascular disease 8% Chronic obstructive pulmonary disease (COPD) 4% Chronic kidney disease 2% Cancer 2%
3. Additional risk factor unique for low-income countries	HIV Tuberculosis Chronic obstructive pulmonary disease (COPD) Rheumatic heart disease (RHD) Cardiomyopathies

The table outlines risk factors for severe COVID-19 disease and co-morbidities from China (*from Guan et al. Clinical characteristics of coronavirus disease 2019 in China, NEJM, 2020*) and low-income settings with a focus on Sub-Saharan Africa.

In case SARS-CoV-2/COVID-19 begins to spread in low-income countries at high risk of airborne diseases, as described above, containment may not be realistic and response efforts will likely need to transition to various mitigation strategies, which could include isolating ill people at home, closing schools, universities, places of religious worship and public events, which would also include attending funerals. Please follow recommendations of leading health organizations as outlined in Table 2.

**Table 2:** Important resources and information.


World Health Organization	www.who.int/health-topics/coronaviru
European Centre for Disease Prevention and Control	www.ecdc.europa.eu/en/coronavirus
US Centers for Disease Control and Prevention	www.cdc.gov/coronavirus/2019-nCoV
Uptodate	www.uptodate.com/contents/coronavirus-disease-2019-covid-19

